# Hepatic tuberculosis induced by rituximab treatment for C1q nephropathy with minimal change disease: a case report

**DOI:** 10.3389/fmed.2025.1621723

**Published:** 2025-08-29

**Authors:** Huifang Wang, Yiqi Huang, Feng Jin, Xinxin Liu

**Affiliations:** ^1^Department of Digestive System, Shaoxing Second Hospital, Shaoxing, Zhejiang, Zhejiang, China; ^2^Department of Nephrology, Shaoxing Second Hospital, Shaoxing, Zhejiang, China; ^3^Department of Functional Examination, Tongde Hospital of Zhejiang Province Afflicted to Zhejiang Chinese Medical University, Hangzhou, Zhejiang, China

**Keywords:** hepatic tuberculosis, rituximab, C1q nephropathy, minimal change disease, case

## Abstract

**Background:**

Rituximab is widely used for autoimmune nephropathy. It depletes B cells, potentially increasing infection risk. Tuberculosis is a rare but severe complication of rituximab treatment. We report a case of liver tuberculosis in a patient with C1q nephropathy with Minimal Change Disease (MCD) treated with rituximab.

**Case presentation:**

In March 2023, an 81-year-old male patient was admitted to Shaoxing Second Hospital with a 2-month history of bilateral lower extremity edema. He was diagnosed with C1q nephropathy with MCD through renal biopsy. After treatment with 2 g rituximab, his proteinuria was relieved. In October 2024, due to B-cell rebound, 0.5 g of rituximab was added. In December 2023, the patient visited our hospital due to a 7-day fever. Abdominal ultrasound revealed a non-uniform hypoechoic liver mass suspected to be an abscess. Empirical antibiotic treatment was ineffective and the condition worsened. A liver biopsy was immediately performed, and the pathology showed characteristic granulomatous inflammation and patchy coagulative necrosis. The patient was ultimately diagnosed with hepatic tuberculosis and received a 1-year anti-tuberculosis treatment, including rifampicin 450 mg qd, isoniazid 300 mg qd, pyrazinamide 1,500 mg qd, and ethambutol 1,000 mg qd. The patient’s temperature returned to normal and abdominal pain was relieved on the third day of treatment. Two months later, a follow-up ultrasound showed a reduction in the left lobe liver mass, and an 8-month CT scan showed complete disappearance of the mass. The patient is currently under follow-up.

**Conclusion:**

Rituximab may be an effective treatment option for C1q nephropathy with MCD. Although the risk of infection with rituximab is relatively low, rare infections such as tuberculosis still need to be vigilant, especially in elderly or immunocompromised patients. Additionally, we recommend routine screening for latent tuberculosis in elderly patients with nephropathy and hypogammaglobulinemia before rituximab treatment.

## 1 Introduction

Rituximab, as a chimeric monoclonal antibody targeting CD20 (1), has been widely used in the treatment of autoimmune kidney diseases such as minimal change disease (MCD), membranous nephropathy, lupus nephritis, and in kidney transplantation (2). It exerts its effect by depleting B cells, but this mechanism may also increase the risk of infection. Schachtner et al. reported that nephropathy patients previously treated with rituximab had higher risks of cytomegalovirus infection, BK virus nephropathy, and severe sepsis (3). Kamar et al. found 9.09% of renal transplant patients died from infections during rituximab treatment (4). Trivin et al. noted 79% of infections in rituximab-treated glomerular disease patients were bacterial, with pneumonia being the most common (5). Tuberculosis, an infectious disease caused by *Mycobacterium tuberculosis*, remains the leading cause of death from infectious diseases globally (6, 7). Tuberculosis represents a rare yet potentially devastating complication in patients undergoing Rituximab treatment, with only sporadic case reports available in the literature (8, 9). To date, it remains uncertain whether rituximab use is associated with an increased risk of tuberculosis. Some studies suggest that Rituximab may lead to the reactivation of latent tuberculosis in kidney disease patients (10, 11); however, the underlying mechanisms and the extent of this risk require further investigation. C1q nephropathy (C1q nephropathy) is a rare glomerular disease characterized by intense C1q deposition in the mesangial area as shown by immunofluorescence staining (12), and it is relatively uncommon in clinical practice. Here, we report a case of liver tuberculosis induced by Rituximab treatment for C1q nephropathy with MCD.

## 2 Case presentation

In March 2023, an 81-year-old male from a low-risk tuberculosis area, with no previous medical or tuberculosis history, was admitted to the Nephrology Department of our hospital due to recurrent bilateral lower extremity edema for 2 months. He had a 6-year history of hypertension and an 8-year history of type 2 diabetes. Upon admission, the physical examination showed a blood pressure of 141/85 mmHg, a body temperature of 37°C, and moderate bilateral lower extremity edema. Laboratory test results were as follows: urine protein: 4+, Urinary Albumin-to-Creatinine Ratio (UACR) ≥ 300 mg/g, 24-h urinary protein (24UP) 4.93 g, albumin (ALB) 18.5 g/L, total cholesterol 10.04 mmol/L, calcium 1.88 mmol/L, serum creatinine (SCR) 71umol/L, hemoglobin (HB) 129 g/L. Tests for *mycobacterium tuberculosis* antibodies, tumor markers, antinuclear antibody (ANA), anti-neutrophil cytoplasmic antibodies (ANCA), and immunofixation electrophoresis were all negative. An abdominal ultrasound revealed that both kidneys were of normal size and shape. Chest CT showed no abnormalities.

The patient was initially diagnosed with nephrotic syndrome and underwent a renal biopsy 3 days after hospital admission. Glomeruli: Light microscopy revealed two glomeruli, one of which was sclerotic. The non-sclerotic glomerulus showed mild focal mesangial proliferation. Electron microscopy demonstrated diffuse podocyte foot process fusion (>80%), segmental basement membrane thickening, and occasional electron-dense deposits in the mesangial areas. Tubulointerstitial: Mild non-specific changes were observed, including tubular epithelial degeneration, focal atrophy, minimal inflammatory cell infiltration, and interstitial fibrosis. Immunofluorescence analysis revealed the following results: IgM (+), C1q (2+), while all other immune complex deposits were negative. Based on the integration of clinical data, light microscopy findings, electron microscopy observations, and immunofluorescence examination, the pathological diagnosis was established as C1q nephropathy with MCD (Supplementary Figure 1). Given the patient’s advanced age, corticosteroid therapy was declined. Additionally, considering that C1q nephropathy typically exhibits a suboptimal response to corticosteroids, Rituximab monotherapy was selected as the treatment regimen (intravenous infusions of 1 g were administered at the 2nd and 4th weeks after admission). Prior to the first and second infusions, the total B cells (CD20+) were 15.5 and 1.8%, respectively. In mid-May, a follow-up measurement revealed CD20 + levels had decreased to 0%. In October 2023, CD20 + was 5.7%, while the 24UP was recorded at 0.3 g. In light of the patient’s remission of proteinuria but the observed rebound in CD20+, an additional dose of Rituximab 0.5 g was administered.

In December 2023, the patient was admitted to our hospital due to a seven-day history of persistent fever. The body temperature was 38.9°C, while all other vital signs were within normal limits. Laboratory findings revealed: white blood cell (WBC) 15.2 × 10^9^/L, neutrophils 75.9%, lymphocytes 15.2%, c-reactive protein (CRP) 25 mg/L, urine protein 1+, 24UP 0.56 g, and ALB 33.5 g/L. Scr, Hb, and procalcitonin (PCT) levels were within normal ranges. Chest CT showed no abnormalities. Abdominal color Doppler ultrasound identified a non-uniform hypoechoic mass measuring 37 × 30 × 32 mm in the medial segment of the left liver lobe. Abdominal contrast-enhanced CT confirmed an irregularly shaped lesion with heterogeneous enhancement in the left lobe of the liver (Supplementary Figure 2).

We suspected it to be liver abscess and upgraded the treatment from 3-day course of piperacillin-tazobactam (4.5 g IV q8d) to meropenem (1 g IV q8d). However, within the following 5 days, the patient’s condition gradually deteriorated, presenting with persistent high fever and abdominal pain, indicating that the simple anti-infective treatment was ineffective. Laboratory re-evaluation revealed a significant deterioration in inflammatory markers. Concurrently, the tuberculin-specific T-cell spot (T-SPOT) test for tuberculosis infection returned positive, whereas serological tests and blood cultures for amoebic infection remained negative, raising the suspicion of tuberculosis. To clarify the diagnosis, a liver biopsy was performed on the ninth day following admission, which demonstrated the presence of characteristic granulomatous inflammatory morphology along with patchy coagulative necrosis (Supplementary Figure 3).

The patient was eventually diagnosed with hepatic tuberculosis and received one-year anti-tuberculosis treatment (rifampicin 450 mg qd, isoniazid 300 mg qd, pyrazinamide 1,500 mg qd, ethambutol 1,000 mg qd). After the anti-tuberculosis treatment began, the patient’s body temperature returned to normal on the third day and abdominal pain was relieved. Two months later, the ultrasound re-examination indicated that the mass in the left lobe of the liver had decreased (13*11 mm), and 8 months later, the CT re-examination showed that the mass had completely disappeared (Supplementary Figure 2). Currently, the patient has stopped the anti-tuberculosis and biological agent treatments. During the treatment period, no adverse drug reactions such as liver function or optic nerve function abnormalities occurred, and regular clinical follow-ups are being conducted. In addition, Supplementary Figure 4 illustrates the timeline of diagnosis and treatment.

## 3 Discussion and conclusion

Rituximab, a monoclonal antibody targeting CD20, is widely utilized in the treatment of various autoimmune diseases (1). While its adverse effects are generally regarded as mild (13), there has been growing concern in recent years regarding its potential association with infectious complications. The precise mechanisms underlying rituximab-induced infections remain incompletely understood. First, rituximab induces prolonged B-cell depletion, leading to reduced antibody production, particularly after repeated dosing, which may result in hypogammaglobulinemia and increase the risk of infection (14). A study in pediatric patients demonstrated that low IgG levels following rituximab treatment were associated with an elevated risk of severe infections, with some patients developing persistent hypogammaglobulinemia (15). Second, rituximab may disrupt the balance of T-cell subsets, impairing cellular immune function and thereby compromising the body’s defense against pathogens (16). Additionally, rituximab may cause delayed neutropenia, further affecting innate immune responses. While some patients may spontaneously recover neutrophil counts, this effect may lead to severe infections, especially in elderly individuals and those with renal failure (17).

The issue of whether rituximab induces reactivation of tuberculosis remains controversial. The Rituximab Consensus Expert Committee and the European Society of Clinical Microbiology and Infectious Diseases Working Group, among others, do not recommend latent tuberculosis screening prior to treatment with CD19- or CD20-targeted monoclonal antibodies (18, 19). In a single-center retrospective analysis, no significant association was observed between rituximab use and tuberculosis or other infections in 56 renal transplant recipients compared to 287 non-recipients (20). A retrospective cohort study involving 60 patients treated with Rituximab for rheumatic diseases indicated that rituximab may be considered as a first-line therapy even in populations at risk for tuberculosis reactivation, particularly in regions with high tuberculosis prevalence and incidence (21). However, scattered case reports describe tuberculosis reactivation in rituximab-treated patients, especially those with a history of prior tuberculosis infection (10). These findings highlight the need for continued vigilance regarding this potential risk. In this case, hepatic tuberculosis developed nine months after Rituximab treatment, an occurrence not previously reported in the literature. Prior to initiating Rituximab therapy, a preliminary infectious disease screening was performed, with no evidence of tuberculosis infection detected. Nevertheless, hepatic tuberculosis emerged following the third administration of Rituximab (cumulative dose of 2.5 g). Serial monitoring of IgG levels revealed a progressive decline, indicative of hypogammaglobulinemia developing during Rituximab treatment. Furthermore, patients with nephrotic syndrome may experience a decrease in protein levels due to hypoproteinemia, which could further enhance the immunosuppressive effect of rituximab. Therefore, the onset of hepatic tuberculosis may be attributed to the compromised immune function commonly observed in elderly patients, compounded by hypogammaglobulinemia induced by Rituximab therapy.

Hepatic tuberculosis represents a rare form of tuberculosis and is typically categorized as extrapulmonary tuberculosis. Despite its varied imaging characteristics, the nonspecific nature of these findings frequently leads to misdiagnosis as other liver pathologies, such as hepatic abscesses or malignancies (22). On imaging studies, hepatic tuberculosis may manifest as multiple small nodular lesions, often associated with central calcifications, bile duct dilation, intrahepatic bile duct stenosis, and liver lobe atrophy (23). While imaging plays a critical role in identifying hepatic tuberculosis lesions, definitive diagnosis relies on pathological examination, particularly confirmation via tissue biopsy (22). Liver biopsy can reveal caseous necrosis and the presence of *Mycobacterium tuberculosis*, which are essential for establishing a diagnosis of hepatic tuberculosis. In this case, we initially detected a liver lesion through imaging examinations. However, empirical anti-infection treatment was ineffective. Eventually, a tissue biopsy confirmed the diagnosis of liver tuberculosis. It is worth noting that this patient has been continuously receiving RTX treatment, and there is no evidence of pulmonary tuberculosis infection either before or after the diagnosis. Therefore, we believe that the liver tuberculosis is directly related to the RTX treatment and do not consider the reactivation of tuberculosis outside the lungs. For treatment, we followed the WHO guidelines on managing *Mycobacterium tuberculosis* infection. Anti-tuberculosis drug dosages for elderly patients should consider age, weight, liver and kidney function, and underlying conditions (24). This patient weighed 60 kg, and the administered doses—rifampicin 450 mg/day, isoniazid 300 mg/day, pyrazinamide 1,500 mg/day, and ethambutol 1,000 mg/day—were all within the adult recommended ranges. No significant adverse effects were observed, so no dose adjustments were made. The 12-month four-drug regimen was selected based on two key factors. First, the patient had hepatic tuberculosis, a form of extrapulmonary tuberculosis, combined with immunosuppression caused by rituximab therapy (resulting in B-cell depletion and hypogammaglobulinemia). Guidelines recommend longer treatment for such cases to reduce recurrence risk. Second, quadruple therapy is the first-line approach, offering broad coverage and synergistic bactericidal effects (25). The patient’s condition improved steadily: the liver mass reduced after 2 months and disappeared completely after 8 months, confirming the effectiveness of the treatment plan. In conclusion, this case involved an elderly patient with C1Q nephropathy, immunosuppression, and liver tuberculosis. After 1 year of four-drug combination therapy, the patient showed favorable outcomes, likely due to early, timely, and adequate anti-tuberculosis treatment.

In 1982, Jones first documented the pathological features of C1q nephropathy (26). In 1985, Jenette and Hipp formally introduced the concept of C1q nephropathy and established its diagnostic criteria (27): diffuse high-intensity C1q deposition in the glomerular mesangial region with an immunofluorescence intensity score of ≥ 2+, while excluding type I membranoproliferative glomerulonephritis, hepatitis B virus-associated glomerulonephritis, and lupus nephritis. Studies have reported that the prevalence of C1q nephropathy is approximately 2.1–6% among pediatric and adult patients undergoing renal biopsy (28, 29), 0.2–2.5% among patients presenting with nephrotic syndrome and persistent proteinuria (27, 30), and 16.5% among adult patients (31). C1q nephropathy has heterogeneous pathological features that can be divided into three main types based on histopathology (32): MCD, focal segmental glomerulosclerosis (FSGS), and immune-mediated proliferative glomerulonephritis. In this case, light microscopy and electron microscopy of the renal biopsy demonstrated diffuse fusion (>80%) of podocyte foot processes with microvillous transformation. Immunofluorescence revealed diffuse C1q (++) deposition in the mesangial area along with IgM deposition. No hypocomplementemia was observed, and serological tests for ANA, ANCA, and hepatitis B virus were negative. These findings support a diagnosis of C1q nephropathy, with renal histology consistent with MCD. The clinical significance and pathological mechanism of C1q deposition in C1q nephropathy have not been fully elucidated: As an initiating component of the classical complement pathway, the deposition in the mesangial area may be related to the binding of IgG and IgM in immune complexes to C1q receptors on mesangial cells after activation of the complement system (33–36). This case’s IgM deposition supports this view, but the mechanism of selective binding of immune complexes is unclear; C1q can bind to polyanionic substances, suggesting that DNA viral infection may be involved in the pathogenesis (37, 38); some studies suggest that the deposition of C1q in minimal change nephropathy may be related to the non-specific trapping of increased plasma proteins in the mesangium (30); the fusion of podocyte foot processes suggests that it may be involved in the pathogenesis. At present, there is a lack of high-quality evidence for effective treatment options for C1q nephropathy. For all patients, glucocorticoids are the initial treatment. Although it is prone to dependence or resistance, the clinical remission rate is approximately 77% (39). For patients who are dependent or resistant to hormones, immunosuppressants such as cyclophosphamide, mycophenolate mofetil, and tacrolimus can be combined. Multiple reports have shown that children with MCD who are treated with prednisone alone are prone to recurrence, dependence, or resistance (40, 41). Combining calcineurin inhibitors can lead to long-term remission and stable renal function (41). In this case, the elderly patient refused hormones and immunosuppressants, and rituximab (375 mg/m^2^ per week, for 4 weeks) was selected, which is consistent with the treatment effect of steroid-dependent cases reported in the literature (42, 43).

In conclusion, rituximab may represent a promising therapeutic option for C1q nephropathy with minor changes. However, an increasing number of case reports highlight that, despite its relatively low infection risk, rituximab use may still be associated with rare infections such as tuberculosis, particularly in elderly patients and those with compromised immune function. Furthermore, this case does not provide sufficient evidence to support routine screening for latent tuberculosis infection in all patients receiving rituximab therapy. Nevertheless, latent tuberculosis infection screening may be considered appropriate for elderly patients with hypogammaglobulinemia.

## Data Availability

The original contributions presented in the study are included in the article/Supplementary material, further inquiries can be directed to the corresponding author.
