# Supplementary material for: Hepatic tuberculosis induced by rituximab treatment for C1q nephropathy with minimal change disease: a case report
Source: Front Med (Lausanne). 2025 Aug 29;12:1621723. doi: 10.3389/fmed.2025.1621723 (PMC12426271; doi:10.3389/fmed.2025.1621723)
Supplement: Supplementary file 1 [file Data_Sheet_1.docx]

Supplementary Material

# Supplementary Figures and Tables

## Supplementary Figures


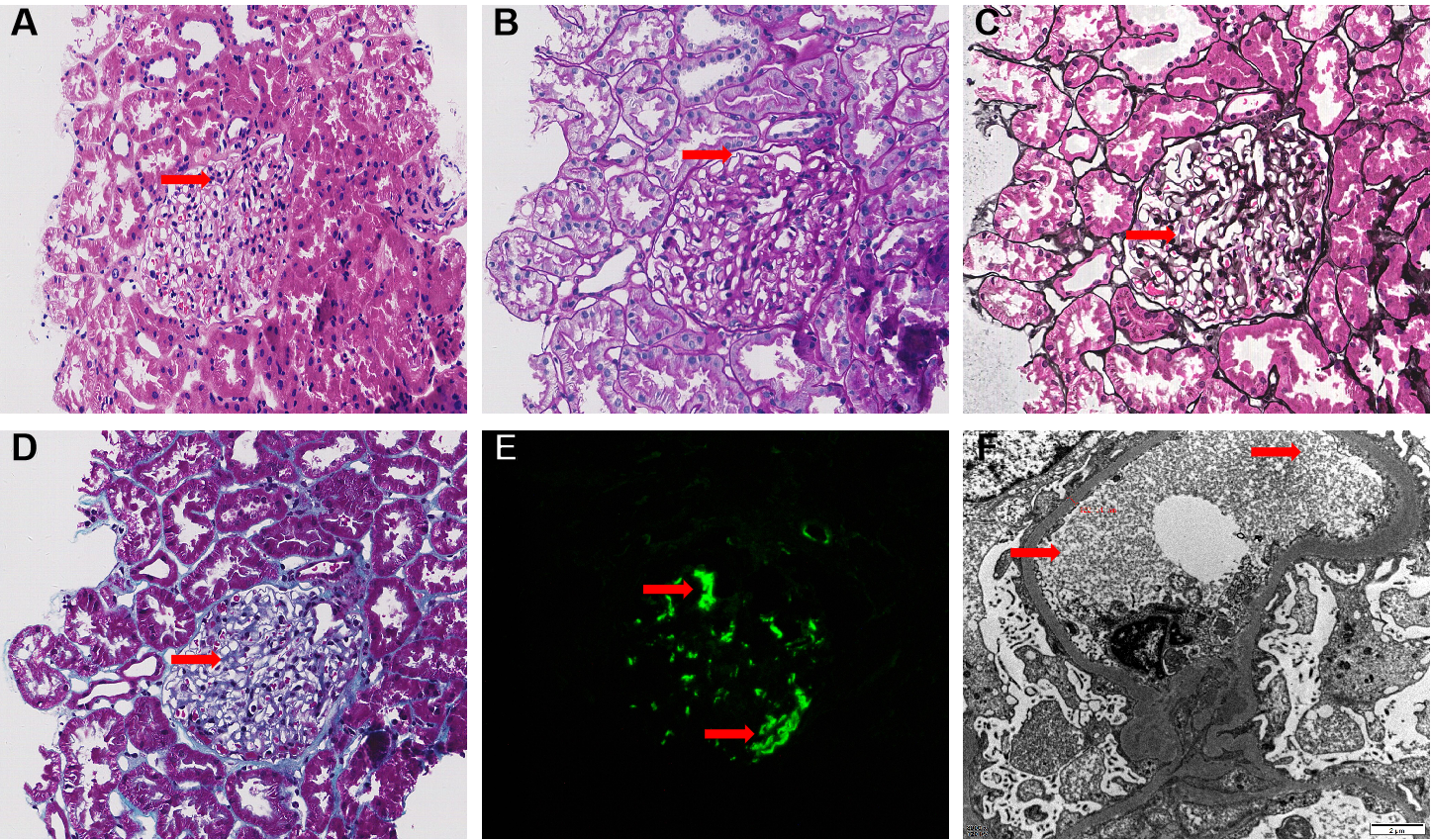


**Supplementary Figure 1.** This patient was diagnosed with C1q nephropathy with MCD by renal biopsy. (A) Hematoxylin-eosin staining showed that the glomerular capillary loops were open well, and occasional inflammatory cell infiltration was observed; focal and segmental mild proliferation of mesangial cells and matrix in the glomerular mesangial area was noted (×200); (B) Periodic acid-Schiff staining showed vacuolar and granular degeneration of renal tubular epithelial cells, and focal and small tubular atrophy (atrophy area approximately 5%); no definite reticulon red protein deposition was observed in all parts of the glomerulus (×400); (C) and (D) Periodic acid silver methenamine staining and masson staining showed no definite reticulon red protein deposition in all parts of the glomerulus (×400); (E) Immunofluorescence showed C1q (2+); (F) Electron microscopy showed scattered electron-dense deposits below the epithelium of the capillary basement membrane, bar = 2 μm.

**
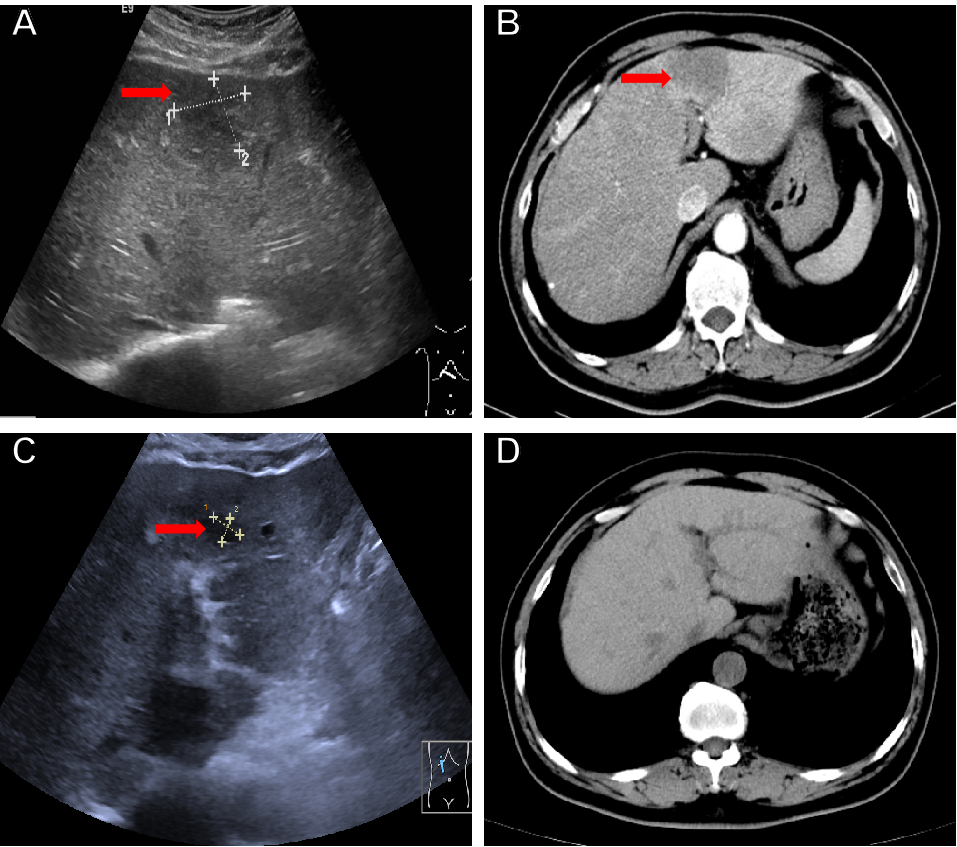
**

**Supplementary Figure 2**. Abdominal ultrasound and CT changes. (A) Abdominal ultrasound revealed a 37 × 30 × 32 mm hypoechoic mass in the medial segment of the left liver lobe; (B) Abdominal CT confirmed an irregular lesion with heterogeneous enhancement in the left liver lobe; (C) Abdominal ultrasound indicated that the mass in the left lobe of the liver had decreased; (D) Abdominal CT showed complete disappearance of the mass.


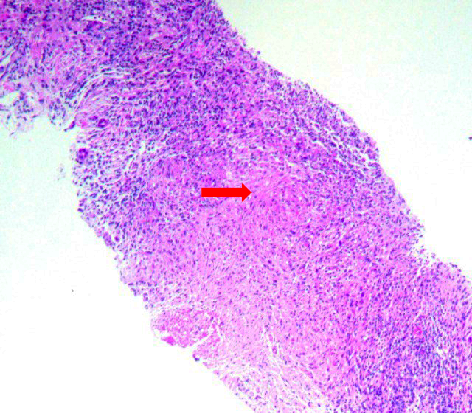


**Supplementary Figure 3**. The liver biopsy showed characteristic granulomatous inflammation and patchy coagulative necrosis (HE × 40)

.


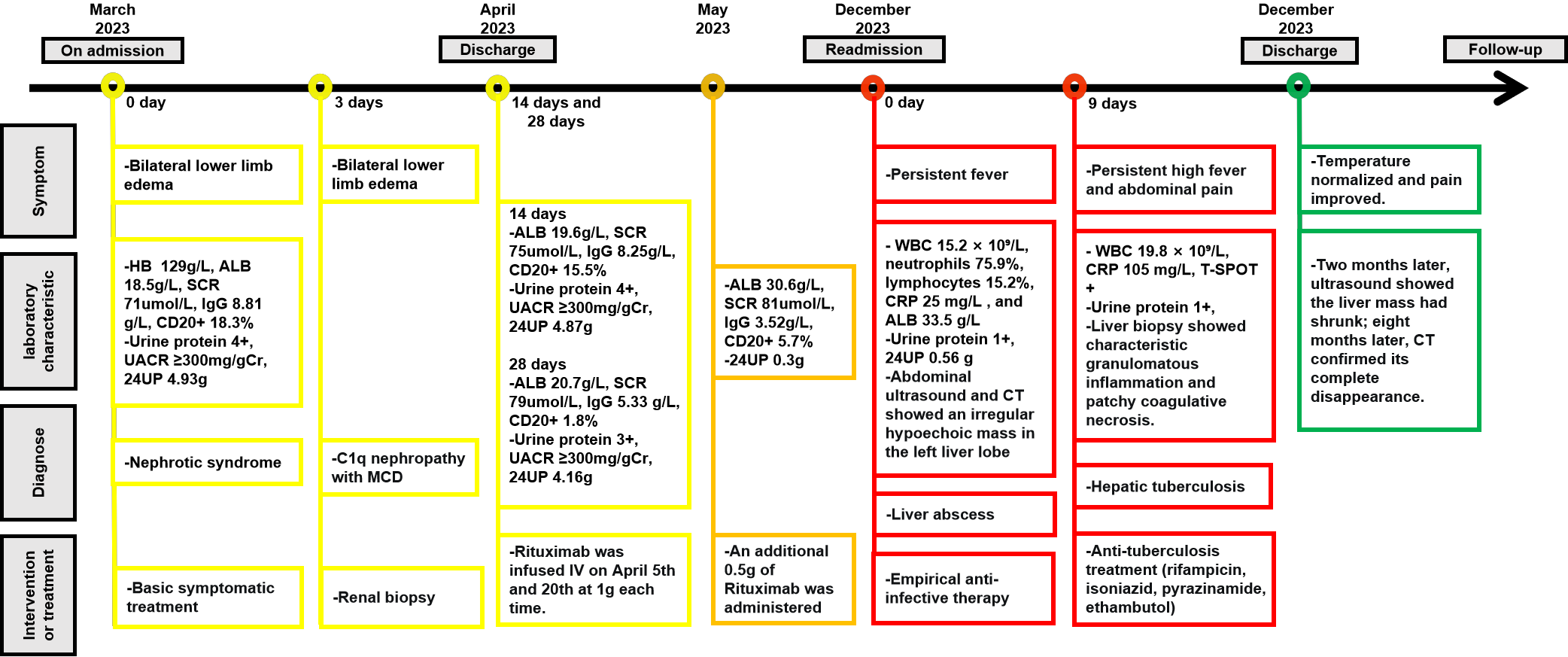


**Supplementary Figure 4**. The timeline for diagnosis and treatment
